# Real-world response rates in patients with human epidermal growth factor receptor 2-positive breast cancer after neoadjuvant treatment using dual human epidermal growth factor receptor 2 blockade

**DOI:** 10.3389/fonc.2026.1895998

**Published:** 2026-09-09

**Authors:** Abdul Shahid Poovathum Parambil, Abul Hussain Mirsa, Azgar Abdul Rasheed, Arun Vasudevan

**Affiliations:** Department of Medical Oncology, KIMS Health, Trivandrum, Kerala, India

**Keywords:** breast neoplasms, injections, intravenous injections, pertuzumab, subcutaneous neoadjuvant therapy, trastuzumab

## Abstract

**Introduction:**

Breast cancer (BC) is the most commonly diagnosed malignancy among women globally and in India. The human epidermal growth factor receptor 2 (HER2)-positive subtype is associated with aggressive disease and poorer prognosis; however, neoadjuvant therapy combining dual HER2 blockade with chemotherapy has been shown to improve pathological complete response (pCR) rates. This study evaluated the real-world effectiveness of dual HER2 blockade in the Indian setting.

**Methods:**

This ambispective, descriptive real-world study was conducted at KIMSHEALTH, Trivandrum, Kerala (1 October 2019–30 April 2025), comprising retrospective (1 October 2019–30 April 2024) and prospective (1 May 2024–30 April 2025) phases. Patients with HER2-positive early BC receiving intravenous (IV) or subcutaneous (SC) pertuzumab and trastuzumab were included. The primary endpoint was pCR. Secondary endpoints included comparison of pCR between the IV and SC groups; assessment of chemotherapy regimen, disease stage, and hormonal status on outcomes; and evaluation of treatment-related toxicities.

**Results:**

A total of 74 patients (35: retrospective; 39: prospective) (mean age: 56.8 ± 9.1 years) were included in the study; 52 (70.3%) received SC, and 22 (29.7%) received IV dual HER2 blockade. The overall pCR rate was 51.4% (SC: 44.2%; IV: 68.2%; p=0.06). Tumor stage (p=0.044) and composite clinical stage (tumor, node, and metastasis) (p=0.019) were significantly associated with pCR in the overall population, whereas estrogen receptor (ER) status was not associated with pCR (p=0.064). Multivariate analysis identified the composite clinical stage as an independent predictor of pCR (odds ratio: 0.242; p=0.012). Anemia was the most common toxicity, followed by hepatic toxicity, with comparable safety between the SC and IV groups.

**Discussion:**

Neoadjuvant dual HER2 blockade in HER2-positive BC is effective and well tolerated in the Indian real-world setting, with tumor stage and composite clinical stage significantly influencing response, while ER status showed no significant association with pCR. SC and IV dual HER2 blockade demonstrated comparable efficacy and safety outcomes. These findings, which are consistent with historical data, further reinforce the therapeutic advancement from trastuzumab-based therapy to dual HER2 blockade, highlighting its improved effectiveness and clinical importance in the neoadjuvant treatment of HER2-positive BC.

## 1 Introduction

Breast cancer (BC) is the leading cancer affecting women and the second most prevalent cancer worldwide (1). Globally, female BC accounted for an estimated 2.3 million new diagnoses and 670,000 deaths in 2022 (2). This global burden is projected to increase, with new cases expected to rise by 38% (to 3.2 million) and deaths by 68% (to 1.1 million) by 2050 (2). According to the GLOBOCAN 2022 estimates, in India, BC ranks first among cancers in women, accounting for 26.6% of all new cancer cases (3). Consistent with these trends, estimates based on data from the National Cancer Registry Programme Report 2020 (which covered cancer incidence data from 28 Population-Based Cancer Registries for the period 2012–2016) indicate that the disease burden of BC will reach approximately 5.6 million disability-adjusted life years by 2025 (4).

The human epidermal growth factor receptor 2 (HER2)-enriched subtype, characterized by overexpression or amplification of the *HER2* gene located on chromosome 17, accounts for approximately 15%–20% of invasive BC cases (5). Compared with HER2-negative disease, HER2 immunohistochemistry (IHC) 3+ and IHC 2+/fluorescence *in situ* hybridization (FISH)-positive BC exhibit more aggressive behavior, with higher rates of cell proliferation and lymph node metastasis and greater resistance to chemotherapy. In the metastatic setting, this subtype demonstrates poor clinical outcomes, including reduced disease-free survival and overall survival rates (6). Importantly, prognosis differs considerably among BC molecular subtypes. Large population-based analyses have identified molecular subtype as an independent prognostic factor, with hormone receptor (HR)-positive [HR+]/HER2-negative tumors generally demonstrating the most favorable outcomes, followed by HR+/HER2-positive and HR-negative [HR-]/HER2-positive tumors, whereas triple-negative BC consistently exhibits the poorest prognosis irrespective of age, race, or stage. Although HER2-positive disease has been associated with aggressive clinical behavior and inferior outcomes compared with HR+/HER2-negative disease, improved outcomes observed in HER2-positive disease may reflect the impact of HER2-targeted therapies (7).

Neoadjuvant therapy (NAT), administered before surgery, is endorsed by major international guidelines, including the 2025 National Comprehensive Cancer Network, the 2021 American Society of Clinical Oncology, and the 2024 European Society for Medical Oncology. It is now the recommended treatment approach for patients with high-risk HER2-positive BC, particularly those with tumors ≥T2 or node-positive disease (8–11). Importantly, achievement of a pathological complete response (pCR) after NAT has been strongly associated with improved event-free and overall survival, especially in HER2-positive disease (12).

Trastuzumab, a monoclonal antibody directed against the HER2 receptor, attaches to its extracellular domain, blocking receptor homodimerization and interrupting HER2-mediated signaling pathways (13). Incorporation of trastuzumab into neoadjuvant chemotherapy has been shown to significantly increase pCR rates among patients with HER2-positive BC. In individuals with HER2-positive locally advanced or inflammatory BC, administration of trastuzumab for 1 year (initiated during the neoadjuvant phase and continued as adjuvant therapy) alongside neoadjuvant chemotherapy improved overall response rates, nearly doubled pCR rates compared with those who did not receive trastuzumab (38% vs. 19%), and lowered the risk of relapse, disease progression, or death (14). Therefore, trastuzumab is a key component of neoadjuvant treatment in HER2-positive early breast cancer (EBC).

Following this, chemotherapy combined with pertuzumab and trastuzumab became the Food and Drug Administration (FDA)-approved NAT for HER2-positive BC (15) supported by robust efficacy and safety demonstrated in pivotal trials, including NeoSphere (pCR rate: 45.8%), TRYPHAENA (pCR rate: 57.3%–66.2%), and BERENICE (pCR rate: approximately 60.7%–61.8%) (16–18). Although pertuzumab and trastuzumab have traditionally been administered via the intravenous (IV) route (19), Phesgo^®^ (pertuzumab/trastuzumab/hyaluronidase-zzxf) is a fixed-dose subcutaneous (SC) formulation that combines pertuzumab and trastuzumab with recombinant human hyaluronidase, enabling delivery of larger drug volumes in a ready-to-use vial (20). Phesgo^®^ received approval from the United States FDA in 2020 and from the Drugs Controller General of India in October 2021. It is indicated for the neoadjuvant treatment of HER2-positive locally advanced, inflammatory, or EBC, as well as for the adjuvant treatment of HER2-positive EBC (21, 22).

Despite demonstrating pharmacokinetic equivalence and comparable efficacy and safety to IV administration (23), along with advantages such as shorter administration time, greater convenience, and reduced invasiveness (24, 25), real-world evidence on the efficacy, safety, and patient-reported outcomes of Phesgo^®^ in the Indian healthcare setting remains limited. The diverse demographic and clinical characteristics of the Indian population highlight the need for a comprehensive real-world assessment of Phesgo^®^, which may provide valuable insights into its benefits and challenges, support informed clinical decision-making, and help shape future policy recommendations.

This study evaluated the real-world effectiveness of dual HER2 blockade with pertuzumab and trastuzumab. The primary objective was to estimate the rate of pCR associated with dual anti-HER2 blockade therapy. Secondary objectives included comparing the effectiveness of SC dual anti-HER2 blockade (Phesgo^®^) with that of IV dual anti-HER2 blockade as assessed by pCR rates; evaluating the influence of specific clinical factors on treatment outcomes; and assessing the toxicity profile associated with dual HER2 blockade.

## 2 Materials and methods

### 2.1 Study design and settings

This ambispective, descriptive, real-world study was conducted at KIMSHEALTH, Trivandrum, Kerala, from 1 October 2019 to 30 April 2025. The retrospective phase spanned from 1 October 2019 to 30 April 2024, and the prospective phase was from 1 May 2024 to 30 April 2025. The study included patients who received dual HER2 blockade with pertuzumab and trastuzumab, administered either intravenously or subcutaneously, for the management of HER2-positive EBC, in accordance with local prescribing information and as part of routine clinical treatment.

### 2.2 Ethical considerations

The study was carried out in compliance with the principles of the Declaration of Helsinki, along with other relevant guidelines, including the Good Clinical Practice Guidelines and those set forth by the Indian Council of Medical Research. As this was a noninterventional study, it was not registered in any publicly accessible clinical trial registry.

The study received approval from the Institutional Ethics Committee (Approval no: KIMS/IHEC/APPROVAL/05/2025/08; Approval date: 31 May 2025). A waiver of consent was obtained for using patient data in research and publication during the retrospective phase of the study. All identifying information was anonymized to ensure patient confidentiality. All participants included in the prospective phase provided written informed consent.

### 2.3 Sample size estimation

The sample size was estimated based on the pCR rate with acceptable precision at a 95% confidence level. Based on prior evidence (26), the expected pCR rate was assumed to be 85.7%. The formula used for the sample size estimation is as follows:


$$n=\frac{Z^{2}\times p\times \left(1-p\right)}{\;d^{2}}$$

where,

*Z* = 1.96 (corresponding to a 95% confidence level).

*p* = 0.857 (assumed to maximize variability and provide a conservative estimate).

*d* = 0.05 (desired margin of error).

Using a Z value of 1.96 and a margin of error of ±8%, the minimum sample size required was estimated to be 74 patients. Given the retrospective real-world nature of the study and the finite number of eligible patients available during the study period, an absolute precision of ±8% was considered acceptable for estimation of the pCR rate in this cohort.

### 2.4 Inclusion criteria

The study included female patients aged 18–75 years with a pathologically confirmed diagnosis of HER2-positive BC and stage II–IIIC disease (T2–T4 with any N, or any T with N1–3, M0), which included locally advanced, inflammatory, or early-stage unilateral and histologically confirmed invasive carcinoma, as well as patients with stage IV oligometastatic BC treated with radical intent. Eligibility also required a primary tumor size of ≥2 cm or node-positive disease, confirmed clinically, radiologically, or by cytology/histopathology. Patients who underwent, or were willing to undergo, definitive surgery (breast-conserving surgery, modified radical mastectomy [MRM], or equivalent surgery) after completing standard neoadjuvant chemotherapy with trastuzumab and pertuzumab were included. Neoadjuvant chemotherapy consisted of six cycles of docetaxel, carboplatin, trastuzumab, and pertuzumab (TCHP) for most patients or four cycles of doxorubicin and cyclophosphamide (AC) followed by four cycles of docetaxel, trastuzumab, and pertuzumab (THP) for a few patients, with a total treatment duration of approximately 18–20 weeks. Patients were assessed for surgery approximately 3–4 weeks after completion of the final chemotherapy cycle. Patients with residual invasive disease following surgery (i.e., those who did not achieve pCR) received adjuvant trastuzumab emtansine (T-DM1) as per standard treatment protocols, based on the KATHERINE trial (27). Additional inclusion criteria were an Eastern Cooperative Oncology Group performance status of 0–2 and receipt of anti-HER2 therapy, including dual HER2 blockade, in accordance with local prescribing information. Patients with oligometastatic BC treated with curative intent were also eligible for inclusion.

### 2.5 Exclusion criteria

Patients were excluded if they had metastatic (M1) disease (except for those with Stage IV oligometastatic disease treated with curative/radical intent), unresectable or inoperable breast carcinoma, or did not undergo surgery for any reason. Exclusion also applied to patients who were nonadherent, did not complete the planned dual HER2 blockade, received treatment inconsistent with local prescribing information, or had incomplete records or missing HR or HER2 status. Additionally, patients with *in situ* carcinoma (ductal carcinoma *in situ* or lobular carcinoma *in situ*), sarcoma, or lymphoma of the breast, as well as those with prior or synchronous malignancies requiring systemic therapy, were excluded.

### 2.6 Data collection

For patients who completed NAT and surgery before 30 April 2024, data were retrospectively extracted from the medical records and the computer database of the institution, whereas for patients enrolled after 1 May 2024, data were collected prospectively. HER2 status was assessed by IHC and FISH **Supplementary Table 1** (8, 28).

In the retrospective phase, information on clinical and pathological treatment responses, the date and type of surgery, and follow-up visit dates were extracted from clinical records, pathology reports, and follow-up documentation. During the prospective phase, these data were collected prospectively throughout the study period.

All data, including outcome measures (such as age), were documented in line with International Agency for Research on Cancer guidelines, and tumors were staged based on the TNM Classification of Malignant Tumours (8th edition). Disease staging was performed according to the institutional protocol using bilateral mammography and positron emission tomography (PET) imaging. Recorded variables included patient age; diagnosis and date of diagnosis; tumor receptor status (estrogen receptor [ER]-positive [ER+] or ER-negative [ER-] and progesterone receptor [PR]-positive [PR+] or PR-negative [PR-]) **Supplementary Table 2** (29); disease stage; chemotherapy backbone; anti-HER2 therapy; dual HER2 blockade with pertuzumab and trastuzumab (IV or SC route); adverse events (including anemia, diarrhea, fatigue, cardiac toxicity, febrile neutropenia, thrombocytopenia, vomiting, lung toxicity, hepatic toxicity, and renal toxicity); and pCR status (pCR or no pCR) **Supplementary Table 3** (30). Treatment-related toxicities were graded according to the Common Terminology Criteria for Adverse Events **Supplementary Table 4** (31).

### 2.7 Study endpoints

The primary endpoint of the study was to evaluate pCR associated with dual HER2 blockade. Secondary endpoints included comparing pCR rates between IV and SC dual HER2 blockade, assessing the impact of the chemotherapy regimen (anthracycline followed by docetaxel, trastuzumab, and pertuzumab [AC → THP] vs. docetaxel, carboplatin, trastuzumab, and pertuzumab [TCHP]), evaluating the effect of disease stage and hormonal status on treatment outcomes, and analyzing the incidence of treatment-related toxicities.

### 2.8 Statistical analysis

Data analysis was performed using the Statistical Package for the Social Sciences (SPSS, version 29.1.0). Descriptive and frequency statistics were used to summarize baseline patient characteristics. Categorical variables are reported as numbers (percentages), while continuous variables are expressed as means with standard deviations (SDs). Associations between categorical variables were assessed using the chi-square test, and comparisons of continuous variables were evaluated using the Student’s t-test. A p value of <0.05 was considered statistically significant.

## 3 Results

A total of 74 patients were included in the study, comprising 35 patients in the retrospective phase and 39 patients in the prospective phase. The mean ± SD age of patients was 56.8 ± 9.1 years. Of these, 52 patients received SC dual HER2 blockade and were assigned to the SC group, whereas 22 patients received IV dual HER2 blockade and were assigned to the IV group.

The demographic and clinical characteristics of the patients are presented in **Table 1**. A majority of patients (44.6%) were in the 51–60 years age group. Approximately 74.3% of patients were postmenopausal. About 83.8% of patients received the TCHP chemotherapy regimen. MRM was the most common breast surgery performed in 71.6% of the overall study population.

**Table 1:** Demographic and clinical characteristics of the overall population, including SC and IV dual HER2 blockade groups.

Parameter	Overall (N = 74)	Dual HER2 blockade (SC) (N = 52)	Dual HER2 blockade (IV) (N = 22)
Age group (years)			
31–40	2 (2.7)	1 (1.9)	1 (4.5)
41–50	16 (21.6)	11 (21.2)	5 (22.7)
51–60	33 (44.6)	22 (42.3)	11 (50.0)
>60	23 (31.1)	18 (34.6)	5 (22.7)
Menopause status			
Postmenopausal	55 (74.3)	41 (78.9)	14 (63.6)
Premenopausal	19 (25.7)	11 (21.1)	8 (36.4)
Chemotherapy regimen			
AC → THP	12 (16.2)	6 (11.5)	6 (27.3)
TCHP	62 (83.8)	46 (88.5)	16 (72.7)
Breast surgery			
BCS	21 (28.4)	18 (34.6)	3 (13.6)
MRM	53 (71.6)	34 (65.4)	19 (86.4)

Data are presented as n (%).

Percentages are calculated with 74 as the denominator.

AC → THP, Anthracycline followed by docetaxel + trastuzumab + pertuzumab; BCS, Breast conservation surgery; HER2, Human epidermal growth factor receptor 2; IV, Intravenous; MRM, Modified radical mastectomy; SC, Subcutaneous; TCHP, Docetaxel + carboplatin + trastuzumab + pertuzumab.

Six patients (8.1%) had oligometastatic disease. Among these patients, the number of metastatic lesions ranged from one to five, with bone being the predominant site of metastasis, followed by liver and lung involvement.

The achievement of pCR in the overall population, including the SC and IV dual HER2 blockade groups, has been presented in **Table 2**. Of the 74 patients included in the study, pCR was achieved in 38 (51.4%). In the SC dual HER2 blockade group, the pCR rate was 44.2% (23 of 52), and in the IV dual HER2 blockade group, it was 68.2% (15 of 22). However, the difference between the groups in achieving pCR was comparable (p=0.06).

**Table 2:** Achievement of pCR in the overall population, including SC and IV dual HER2 blockade groups.

pCR achieved	Overall (N = 74)	Dual HER2 blockade (SC) (N = 52)	Dual HER2 blockade (IV) (N = 22)
Yes	38 (51.4)	23 (44.2)	15 (68.2)
No	36 (48.6)	29 (55.8)	7 (31.8)

HER2, Human epidermal growth factor receptor 2; IV, Intravenous; pCR, Pathological complete response; SC, Subcutaneous.

Among the clinical factors analyzed across the entire cohort, only tumor stage (p=0.044) and composite clinical stage (tumor, node, and metastasis) (p=0.019) were significantly associated with achievement of pCR. Other factors, including age (p=0.789), menopausal status (p=0.6923), nodal stage (p=0.254), tumor grade (p=0.812), ER status (p=0.064), progesterone (PR) status (p=0.064), chemotherapy regimen (p=0.999), Kiel 67 (Ki-67) index (p=0.380), and oligometastases (p=0.424), did not show a statistically significant association with pCR **Table 3**.

**Table 3:** Association between clinical factors and pCR in overall patients.

Parameter	pCR		Overall	p value
	Yes	No		
Age (years)				
≤50	10 (13.5)	8 (10.8)	18 (24.3)	0.789
>50	28 (37.8)	28 (37.8)	56 (75.7)	
Menopausal status				
Postmenopausal	27 (36.5)	28 (37.8)	55 (74.3)	0.6923
Premenopausal	11 (14.9)	8 (10.8)	19 (25.7)	
Tumor stage				
T1	2 (2.7)	1 (1.4)	3 (4.1)	**0.044**
T2	25 (33.8)	14 (18.9)	39 (52.7)	
T3	5 (6.7)	6 (8.1)	11 (14.8)	
T4	6 (8.1)	15 (20.3)	21 (28.4)	
Nodal stage				
N0	5 (6.8)	2 (2.7)	7 (9.5)	0.254
N1	26 (35.1)	20 (27.0)	46 (62.1)	
N2	4 (5.4)	8 (10.8)	12 (16.2)	
N3	3 (4.1)	6 (8.1)	9 (12.2)	
Composite clinical stage				
I–II	25 (33.7)	13 (17.6)	38 (51.3)	**0.019**
III–IV	13 (17.6)	23 (31.1)	36 (48.7)	
Grade				
2	24 (32.4)	21 (28.4)	45 (60.8)	0.812
3	14 (18.9)	15 (20.3)	29 (39.2)	
ER status				
Positive	18 (24.3)	25 (33.8)	43 (58.1)	0.064
Negative	20 (27.0)	11 (14.9)	31 (41.9)	
PR status				
Positive	18 (24.3)	25 (33.8)	43 (58.1)	0.064
Negative	20 (27.0)	11 (14.9)	31 (41.9)	
Chemotherapy regimen				
AC → THP	6 (8.1)	6 (8.1)	12 (16.2)	0.999
TCHP	32 (43.3)	30 (40.5)	62 (83.8)	
Ki-67 index* (%)				
<20	1 (1.4)	2 (2.8)	3 (4.2)	0.380
≥20	34 (47.9)	34 (47.9)	68 (95.8)	
Oligometastases				
Yes	2 (2.7)	4 (5.4)	6 (8.1)	0.424
No	36 (48.7)	32 (43.2)	68 (91.9)	

Data are presented as n (%).

Percentages are calculated with 74 as the denominator.

*Values have been calculated from data available for 71 patients, with 71 as the denominator.

AC → THP, Anthracycline followed by docetaxel + trastuzumab + pertuzumab; ER, Estrogen receptor; Ki-67, Kiel 67; pCR, Pathological complete response; PR, Progesterone receptor; TCHP, Docetaxel + carboplatin + trastuzumab + pertuzumab.Bold values indicate statistically significant differences (p<0.05).

With respect to the SC dual HER2 blockade group, only the composite clinical stage showed a statistically significant association with pCR rates (p=0.0108). No statistically significant associations were observed for other clinical factors, including age (p=0.329), menopausal status (p=0.1822), ER status (p=0.2242), and Ki-67 index (p=0.4969) **Supplementary Table 5**. In the IV dual HER2 blockade group, none of the evaluated clinical factors, including age (p=0.334), menopausal status (p=0.3426), composite clinical stage (p=0.9999), ER status (p=0.6517), and Ki-67 index (p=0.999), were significantly associated with achieving pCR **Supplementary Table 5**.

**Table 4** presents the multivariate analysis of clinical factors associated with pCR. Composite clinical stage emerged as a statistically significant factor, with patients in stages III and IV having lower odds of achieving pCR compared with those in stages I and II (odds ratio=0.242, 95% confidence interval: 0.080–2.732; p=0.012). Other clinical factors, including age, tumor grade, ER status, PR status, chemotherapy regimen, Ki-67 index, and route of administration, were not statistically significantly associated with achieving pCR (p>0.05 for all).

**Table 4:** Multivariate logistic regression models predicting total pCR in overall patients.

Parameter	OR (95% CI)	p value
Age (≤50 vs. >50 years)	1.578 (0.439–5.675)	0.485
Composite clinical stage (III–IV vs. I–II)	0.242 (0.080–0.732)	0.012
Grade (3 vs. 2)	0.965 (0.330–2.824)	0.948
ER (positive vs. negative)	0.661 (0.217–2.613)	0.466
PR (positive vs. negative)	0.784 (0.230–2.669)	0.697
Chemotherapy regimen (TCHP vs. AC → THP)	2.433 (0.444–13.325)	0.305
Ki-67 index (%) (≥20 vs.<20)	1.561 (0.120–29.168)	0.733
Route of administration (SC vs. IV)	0.487 (0.143–1.658)	0.250
Constant	0.821	0.943

AC → THP, Anthracycline followed by docetaxel + trastuzumab + pertuzumab; CI, Confidence interval; ER, Estrogen receptor; IV, Intravenous; Ki-67, Kiel 67; OR, Odds ratio; pCR, Pathologic complete response; PR, Progesterone receptor; SC, Subcutaneous; TCHP, Docetaxel + carboplatin + trastuzumab + pertuzumab.

In the overall cohort, the most frequently reported toxicity was anemia (95.9%), followed by hepatic toxicity (73.0%). Other commonly observed toxicities included thrombocytopenia (50.0%), diarrhea (48.6%), neutropenia (29.7%), fatigue (21.6%), vomiting (8.1%), renal toxicity (5.4%), lung toxicity (2.7%), and cardiac toxicity (1.4%). Details of toxicity profiles by grade in the SC and IV dual HER2 blockade groups are presented in **Table 5**, with most toxicities in the overall population being grade 1 or grade 2. The incidence of anemia (p=0.532), neutropenia (p=0.6711), thrombocytopenia (p=0.7764), diarrhea (p=0.999), fatigue (p=0.7692), vomiting (p=0.9999), lung toxicity (p=0.9999), and hepatic toxicity (p=0.4701) was comparable between the SC and IV dual HER2 blockade groups **Table 5**.

**Table 5:** Comparison of toxicities in patients by treatment group [dual HER2 blockade (SC) vs. dual HER2 blockade (IV)].

Parameter	Dual HER2 blockade (SC)	Dual HER2 blockade (IV)	Overall	p value
Anemia	51 (68.9)	20 (27.0)	71 (95.9)	
Grade 1	18 (24.3)	7 (9.5)	25 (33.8)	0.532
Grade 2	26 (35.1)	10 (13.5)	36 (48.6)	
Grade 3	7 (9.5)	2 (2.7)	9 (12.2)	
Grade 4	0 (0)	1 (1.4)	1 (1.4)	
Neutropenia	12 (16.2)	10 (13.5)	22 (29.7)	
Grade 1	6 (8.1)	4 (5.4)	10 (13.5)	0.6711
Grade 2	3 (4.1)	5 (6.8)	8 (10.8)	
Grade 3	2 (2.7)	0 (0)	2 (2.7)	
Grade 4	1 (1.4)	1 (1.4)	2 (2.7)	
Thrombocytopenia	27 (36.5)	10 (13.5)	37 (50.0)	
Grade 1	15 (20.3)	8 (10.8)	23 (31.1)	0.7764
Grade 2	8 (10.8)	2 (2.7)	10 (13.5)	
Grade 3	2 (2.7)	0 (0)	2 (2.7)	
Grade 4	2 (2.7)	0 (0)	2 (2.7)	
Diarrhea	30 (40.5)	6 (8.1)	36 (48.6)	
Grade 1	16 (21.6)	3 (4.1)	19 (25.7)	0.999
Grade 2	11 (14.9)	2 (2.7)	13 (17.6)	
Grade 3	3 (4.1)	1 (1.4)	4 (5.5)	
Fatigue	12 (16.2)	4 (5.4)	16 (21.6)	
Grade 1	3 (4.1)	2 (2.7)	5 (6.8)	0.7692
Grade 2	6 (8.1)	2 (2.7)	8 (10.8)	
Grade 3	3 (4.1)	0 (0)	3 (4.1)	
Vomiting	4 (5.4)	2 (2.7)	6 (8.1)	
Grade 1	3 (4.1)	2 (2.7)	5 (6.8)	0.9999
Grade 2	1 (1.4)	0 (0)	1 (1.4)	
Lung toxicity	1 (1.4)	1 (1.4)	2 (2.7)	
Grade 1	1 (1.4)	0 (0)	1 (1.4)	0.9999
Grade 3	0 (0)	1 (1.4)	1 (1.4)	
Cardiac toxicity	0 (0)	1 (1.4)	1 (1.4)	
Grade 3	0 (0)	1 (1.4)	1 (1.4)	NA
Hepatic toxicity	37 (50.0)	17 (23.0)	54 (73.0)	
Grade 1	32 (43.2)	14 (18.9)	46 (62.2)	0.4701
Grade 2	4 (5.4)	1 (1.4)	5 (6.8)	
Grade 3	1 (1.4)	2 (2.7)	3 (4.1)	
Renal toxicity	3 (4.1)	1 (1.4)	4 (5.4)	
Grade 1	3 (4.1)	1 (1.4)	4 (5.4)	NA

Data are presented as n (%).

Percentages are calculated with 74 as the denominator.

HER2, Human epidermal growth factor receptor 2; IV, Intravenous; NA, Not applicable; SC, Subcutaneous.Bold values represent the overall number and percentage of patients experiencing each toxicity, irrespective of toxicity grade. Grade-specific values are presented separately below each toxicity.

In the context of the risk of occurrence of toxicities between the SC and IV dual HER2 blockade groups, the risk of experiencing diarrhea was statistically significantly higher in patients receiving SC dual HER2 blockade than in those receiving IV dual HER2 blockade (risk ratio=2.1154, 95% CI: 1.0287–4.3502; p=0.0224). The risks of anemia, thrombocytopenia, fatigue, and renal toxicity were higher in patients receiving SC dual HER2 blockade than in those receiving IV dual HER2 blockade, while the risks of neutropenia, vomiting, lung toxicity, cardiac toxicity, and hepatic toxicity were lower in the SC dual HER2 blockade group compared with those in the IV dual HER2 blockade group. However, the risk of experiencing these toxicities was comparable (p>0.05).

## 4 Discussion

This study evaluated the effectiveness of dual HER2 blockade with pertuzumab and trastuzumab in the real-world setting in India, generating comprehensive real-world evidence on Phesgo^®^ to support its broader adoption in the management of HER2-positive BC in India.

The overall pCR rate was 51.4%; pCR was achieved in 44.2% of patients receiving SC dual HER2 blockade and in 68.2% of those receiving IV dual HER2 blockade. Tumor stage and composite clinical stage were significantly associated with pCR in the overall population, whereas only the composite clinical stage was significantly associated with pCR in the SC dual HER2 blockade group. The composite clinical stage was statistically significant in multivariate analysis, with patients in stage III–IV having lower odds of achieving pCR compared with those in stage I–II. Anemia was the most frequently reported toxicity in the overall population, followed by hepatic toxicity. Most toxicities were grade 1 or 2, and toxicity profiles were comparable between the SC and IV dual HER2 blockade groups.

The overall pCR rate of 51.4% observed in the present study, irrespective of the route of administration, is consistent with previously reported pCR rates for neoadjuvant dual HER2 blockade combined with chemotherapy in HER2-positive EBC. When stratified by route of administration, the pCR rate was higher in the IV dual HER2 blockade group (68.2%) than in the SC group (44.2%); however, the difference between the groups was comparable.

With respect to IV dual HER2 blockade, the pCR rate of 68.2% observed in the present study was higher than that reported in major neoadjuvant trials, including FeDeriCa and NeoSphere. In the phase III FeDeriCa study, which compared an SC fixed-dose combination of pertuzumab and trastuzumab with IV pertuzumab plus trastuzumab in 500 patients with HER2-positive EBC, the pCR rate in the IV group was 59.5% (23). In the NeoSphere trial, the pCR rate for the IV dual HER2 blockade arm (IV pertuzumab + trastuzumab + docetaxel) in the neoadjuvant setting was 45.8%, demonstrating the efficacy of dual HER2 blockade in combination with chemotherapy (16).

The FeDeriCa study reported comparable pCR rates between the SC and IV groups (59.7% and 59.5%, respectively), confirming similar efficacy between the two routes of administration (23). This finding aligns with the present study. In contrast, a Japanese real-world study reported significantly higher pCR rates with SC dual HER2 blockade compared with those with IV therapy (77.4% vs. 49.3%; p=0.008) (32).

The lower pCR rate observed in the SC group in this study may be due to several factors, including a small and uneven sample size, differences in patient demographics and baseline disease characteristics, and variations in the chemotherapy backbone used.

Dual HER2 blockade works through the combined action of trastuzumab and pertuzumab, which target distinct epitopes on the HER2 receptor. Trastuzumab restricts HER2 dimerization, while pertuzumab inhibits heterodimerization of HER2 with other HER family receptors (particularly human epidermal growth factor receptor 3). This combination prevents HER2 activation and downstream signaling, enhances antitumor activity via antibody-dependent cellular cytotoxicity, and may also help decrease drug resistance (33).

The present study evaluated the association between various clinical factors and pCR in the overall patient population. Among the factors analyzed, tumor stage and composite clinical stage were the only variables significantly associated with pCR, with the highest pCR rates observed in T2 tumors and lower pCR rates in patients with composite clinical stage III–IV disease. These findings are consistent with existing literature, which demonstrates that lower tumor stages are significantly associated with higher pCR rates (34), whereas higher composite clinical stages are associated with lower pCR rates (35).

Interestingly, age, menopausal status, nodal stage, tumor grade, chemotherapy regimen, Ki-67 index, and oligometastases were not significantly associated with pCR in the overall patient population. These findings are consistent with a single-institution study reporting no significant associations between pCR rates and age, menopausal status, clinical N stage, or Ki-67 level (36). Similarly, another analysis showed that tumor grade, menopausal status, Ki-67, and age at diagnosis did not predict pCR (37). In line with these observations, a large study of women with stage I–III BC demonstrated no significant differences in pCR rates across age groups (≤40, 41–60, and ≥61 years); notably, no age-related differences in pCR were observed among patients with the HER2 subtype (38). Although ER status did not reach statistical significance (p=0.064), ER- tumors (20 achieved pCR among 31 patients; 64.5%) demonstrated a numerically higher pCR rate than ER+ tumors (18 achieved pCR among 43 patients; 41.9%), representing a clinically meaningful difference. The absence of statistical significance may reflect the limited sample size and consequent limited statistical power to detect moderate effect sizes. Notably, this trend aligns with findings from major neoadjuvant HER2-positive BC studies, including NeoSphere, TRYPHAENA, and the CTNeoBC pooled analysis, which have consistently reported higher pCR rates among HER2-positive/HR- tumors than among HER2-positive/HR+ tumors (16, 17, 39).

In the present study, pCR was achieved in 49.1% (27 of 55) of postmenopausal patients and 57.9% (11 of 19) of premenopausal patients; however, menopausal status was not significantly associated with pCR (p=0.6923). Consistent with these results, a real-world study from China involving patients with HER2-positive BC receiving neoadjuvant dual anti-HER2 therapy plus chemotherapy reported comparable pCR rates in postmenopausal (60.0%) and premenopausal (62.2%) patients, with no significant association between menopausal status and pCR (p=0.770) (36).

In the present study, pCR was not significantly influenced by the chemotherapy regimen in the overall patient population. In contrast, Yang et al. reported that chemotherapy regimens similar to those used in the present study (TCHP and AC → THP) were independent predictors of pCR in the overall cohort (p<0.001 for TCHP and p=0.014 for AC → THP) (40). Similarly, another study from China demonstrated that treatment regimen was an independent predictor of total pCR (p=0.005), with higher proportions of patients achieving pCR in both the TCHP (73.1% vs. 26.9%) and AC → THP (65.1% vs. 34.9%) groups compared with those who did not achieve pCR (41). The discrepancy between the present study and these reports may be attributable to the higher proportion of HR+ patients in this cohort, in whom pCR appears to be less influenced by chemotherapy regimen. Consistent with this, Ma et al. reported higher pCR rates in a population with a greater proportion of HR- patients, who are known to exhibit greater sensitivity to therapy (41). Reduced pCR rates in HER2-positive/HR+ tumors have been associated with lower HER2 dependence and increased ER pathway activity, whereas HER2-positive/HR- tumors are more HER2-driven and therefore more responsive to treatment (16, 17, 42, 43). Overall, these findings suggest that, within this cohort, the chemotherapy regimen did not significantly influence pCR, likely due to the predominance of HR+ tumors. This underscores the importance of tumor biology alongside treatment factors when predicting response to dual HER2 blockade.

In the present study, anemia (95.9%) was the most frequently reported toxicity in the overall population, followed by hepatic toxicity (73.0%), thrombocytopenia (50.0%), diarrhea (48.6%), neutropenia (29.7%), fatigue (21.6%), vomiting (8.1%), renal toxicity (5.4%), lung toxicity (2.7%), and cardiac toxicity (1.4%). Most adverse events were mild to moderate in severity, with grade 1 and 2 toxicities predominating, while grade 3 and 4 events were less common. The overall incidence of toxicities was comparable between the SC and IV dual HER2 blockade groups. Consistent with the present findings, the FeDeriCa trial reported similar safety profiles for IV and SC dual HER2 blockade in patients with HER2-positive EBC, with anemia as the most common hematologic toxicity in both the IV (42.5%) and SC (33.9%) groups. Most adverse events were grade 1 or 2 in severity (23). Grade 3 or 4 anemia is commonly associated with carboplatin-containing regimens such as TCHP, with platinum-induced anemia attributed to direct suppression of erythroid progenitor cells in the bone marrow and nephrotoxic effects on erythropoietin-producing cells in the kidney (44). Collectively, the predominance of low-grade toxicities and the comparable safety profiles support the overall safety of dual HER2 blockade with both IV and SC administration.

As a real-world observational study, this analysis has limitations, including potential selection bias and confounding factors such as differences in patient age, tumor characteristics, and treatment regimens, which may have influenced both the choice of administration route and treatment outcomes in the absence of randomization. The generalizability of the findings is further limited by the single-center design, which may not fully represent broader patient populations due to variations in access to healthcare facilities and willingness to participate. Additionally, the small sample size may have influenced the observed associations between clinical variables and pCR. Furthermore, the relatively limited number of pCR events in relation to the number of predictors included in the multivariable analysis may have increased the risk of model overfitting and instability of the estimated odds ratios.

Over half of the patients in the study achieved pCR. Tumor stage and composite clinical stage emerged as statistically significant determinants of pCR in the overall cohort of dual HER2 blockade, highlighting their potential relevance in guiding treatment decisions. The incidence of toxicities was comparable between the SC and IV dual HER2 blockade groups, demonstrating similar safety profiles and indicating that dual HER2 targeting is effective and generally well tolerated in real-world settings. These findings, consistent with historical data, further reinforce the therapeutic advancement from trastuzumab-based therapy to dual HER2 blockade, highlighting its improved effectiveness and clinical importance in the neoadjuvant treatment of HER2-positive BC.

## Data Availability

The original contributions presented in the study are included in the article/**Supplementary Material**. Further inquiries can be directed to the corresponding author.

## References

[1] International Agency for Research on Cancer (IARC) World Health Organization . Breast Cancer Cases and Deaths are Projected to Rise Globally (2025). Available online at: https://www.iarc.who.int/wp-content/uploads/2025/02/pr361_E.pdf (Accessed July 2, 2025).

[2] Kim J Harper A McCormack V . Global patterns and trends in breast cancer incidence and mortality across 185 countries. Nat Med. (2025) 31:1154–62. doi: 10.1038/s41591-025-03502-3 39994475

[3] Ferlay J Ervik M Lam F Laversanne M Colombet M Mery L . Global Cancer Observatory: Cancer Today (2024). Available online at: https://gco.iarc.who.int/today (Accessed July 2, 2025).

[4] Kulothungan V Ramamoorthy T Sathishkumar K . Burden of female breast cancer in India: estimates of YLDs, YLLs, and DALYs at national and subnational levels based on the national cancer registry programme. Breast Cancer Res Treat. (2024) 205:323–32. doi: 10.1007/s10549-024-07264-3 38433127 PMC11101532

[5] Ahn S Woo JW Lee K Park SY . HER2 status in breast cancer: changes in guidelines and complicating factors for interpretation. J Pathol Transl Med. (2020) 54:34–44. doi: 10.4132/jptm.2019.11.03 31693827 PMC6986968

[6] O’Shaughnessy J Gradishar W O’Regan R Gadi V . Risk of recurrence in patients with HER2+ early-stage breast cancer: literature analysis of patient and disease characteristics. Clin Breast Cancer. (2023) 23:350–62. doi: 10.1016/j.clbc.2023.03.007 37149421

[7] Hwang KT Kim J Jung J Chang JH Chai YJ Oh SW . Impact of breast cancer subtypes on prognosis of women with operable invasive breast cancer: a population-based study using SEER database. Clin Cancer Res. (2019) 25:1970–9. doi: 10.1158/1078-0432.ccr-18-2782 30559169

[8] NCCN Clinical Practice Guidelines in Oncology . Breast Cancer Version 4.2025 (2025). Available online at: https://www.nccn.org/guidelines/guidelines-detail?category=1&id=1419 (Accessed July 31, 2018).

[9] Korde LA Somerfield MR Carey LA . Neoadjuvant chemotherapy, endocrine therapy, and targeted therapy for breast cancer: ASCO guideline. J Clin Oncol. (2021) 39:1485–505. doi: 10.1200/jco.20.03399 33507815 PMC8274745

[10] Loibl S André F Bachelot T . Early breast cancer: ESMO clinical practice guideline for diagnosis, treatment and follow-up. Ann Oncol. (2024) 35:159–82. doi: 10.1016/j.annonc.2023.11.016 38101773

[11] Dowling GP Keelan S Toomey S Daly GR Hennessy BT Hill ADK . Review of the status of neoadjuvant therapy in HER2-positive breast cancer. Front Oncol. (2023) 13:1066007. doi: 10.3389/fonc.2023.1066007 36793602 PMC9923093

[12] Spring LM Fell G Arfe A . Pathologic complete response after neoadjuvant chemotherapy and impact on breast cancer recurrence and survival: a comprehensive meta-analysis. Clin Cancer Res. (2020) 26:2838–48. doi: 10.1158/1078-0432.ccr-19-3492 32046998 PMC7299787

[13] Greenblatt K Trastuzumab KK . In: Statpearls (2024). Treasure Island (FL: StatPearls Publishing. Available online at: https://www.ncbi.nlm.nih.gov/books/NBK532246/ (Accessed July 2, 2025).

[14] Gianni L Eiermann W Semiglazov V . Neoadjuvant chemotherapy with trastuzumab followed by adjuvant trastuzumab versus neoadjuvant chemotherapy alone, in patients with HER2-positive locally advanced breast cancer (the NOAH trial): a randomised controlled superiority trial with a parallel HER2-negative cohort. Lancet. (2010) 375:377–84. doi: 10.1016/s0140-6736(09)61964-4 20113825

[15] Amiri-Kordestani L Wedam S Zhang L . First FDA approval of neoadjuvant therapy for breast cancer: pertuzumab for the treatment of patients with HER2-positive breast cancer. Clin Cancer Res. (2014) 20:5359–64. doi: 10.1158/1078-0432.ccr-14-1268 25204553

[16] Gianni L Pienkowski T Im YH . Efficacy and safety of neoadjuvant pertuzumab and trastuzumab in women with locally advanced, inflammatory, or early HER2-positive breast cancer (NeoSphere): a randomised multicentre, open-label, phase 2 trial. Lancet Oncol. (2012) 13:25–32. doi: 10.1016/s1470-2045(11)70336-9 22153890

[17] Schneeweiss A Chia S Hickish T . Pertuzumab plus trastuzumab in combination with standard neoadjuvant anthracycline-containing and anthracycline-free chemotherapy regimens in patients with HER2-positive early breast cancer: a randomized phase II cardiac safety study (TRYPHAENA. Ann Oncol. (2013) 24:2278–84. doi: 10.1093/annonc/mdt182 23704196

[18] Swain SM Ewer MS Viale G . Pertuzumab, trastuzumab, and standard anthracycline- and taxane-based chemotherapy for the neoadjuvant treatment of patients with HER2-positive localized breast cancer (BERENICE): a phase II, open-label, multicenter, multinational cardiac safety study. Ann Oncol. (2018) 29:646–53. doi: 10.1093/annonc/mdx773 29253081 PMC5888999

[19] Ziegengeist JL Tan AR . A clinical review of subcutaneous trastuzumab and the fixed-dose combination of pertuzumab and trastuzumab for subcutaneous injection in the treatment of HER2-positive breast cancer. Clin Breast Cancer. (2025) 25:e124–32. doi: 10.1016/j.clbc.2024.10.005 39567339

[20] Jagosky M Tan AR . Combination of pertuzumab and trastuzumab in the treatment of HER2-positive early breast cancer: a review of the emerging clinical data. Breast Cancer. (2021) 13:393–407. doi: 10.2147/bctt.s176514 34163239 PMC8213954

[21] Central Drugs Standard Control Organisation . CDSCO Approved Drugs/Vaccines/R-DNA/Blood Product. Available online at: https://cdscoonline.gov.in/CDSCO/cdscoDrugs (Accessed September 17, 2025).

[22] Phesgo® Product Information (2024). Available online at: https://www.accessdata.fda.gov/drugsatfda_docs/label/2024/761170s007lbl.pdf (Accessed July 3, 2025).

[23] Tan AR Im SA Mattar A . Fixed-dose combination of pertuzumab and trastuzumab for subcutaneous injection plus chemotherapy in HER2-positive early breast cancer (FeDeriCa): a randomised, open-label, multicentre, non-inferiority, phase 3 study. Lancet Oncol. (2021) 22:85–97. doi: 10.1016/s1470-2045(20)30536-2 33357420

[24] DuMond B Patel V Gross A Fung A Weber S . Fixed-dose combination of pertuzumab and trastuzumab for subcutaneous injection in patients with HER2-positive breast cancer: a multidisciplinary approach. J Oncol Pharm Pract. (2021) 27:1214–21. doi: 10.1177/1078155221999712 33719721

[25] Waks AG Chen EL Graham N . Subcutaneous vs intravenous trastuzumab/pertuzumab: a time and motion substudy of a phase II trial of adjuvant trastuzumab/pertuzumab for stage I HER2+ breast cancer (ADEPT trial. JCO Oncol Pract. (2025) 21:351–7. doi: 10.1200/op.24.00021 39028923 PMC11925349

[26] Yanagihara K Yoshida M Awaji K . Real-world outcomes of subcutaneous PHESGO® in HER2-positive breast cancer: pathological response, sequencing, and safety. Curr Oncol. (2025) 32:658. doi: 10.3390/curroncol32120658 41440186 PMC12732124

[27] von Minckwitz G Huang CS Mano MS Loibl S Mamounas EP Untch M . Trastuzumab emtansine for residual invasive HER2-positive breast cancer. N Engl J Med. (2019) 380:617–28. doi: 10.1056/nejmoa1814017 30516102

[28] Wolff AC Hammond MEH Allison KH . Human epidermal growth factor receptor 2 testing in breast cancer: American Society of Clinical Oncology/College of American Pathologists clinical practice guideline focused update. J Clin Oncol. (2018) 36:2105–22. doi: 10.5858/arpa.2018-0902-sa 29846122

[29] National Cancer Institute . Er (Estrogen Receptor) Total Allred Score. Bethesda: National Cancer Institute (2025).

[30] Choudhary P Gogia A Deo SVS . Correlation of pathological complete response with outcomes in locally advanced breast cancer treated with neoadjuvant chemotherapy: an ambispective study. Cancer Research Statistics Treat. (2021) 4:611–20. doi: 10.4103/crst.crst_197_2141514234

[31] National Cancer Institute . Common Terminology Criteria for Adverse Events (CTCAE (2025). Available online at: https://dctd.cancer.gov/research/ctep-trials/for-sites/adverse-events (Accessed November 18, 2025).

[32] Kinoshita H Kashiwagi S Nishikawa M . Efficacy and safety of subcutaneous pertuzumab-trastuzumab (Phesgo) in neoadjuvant treatment of HER2-positive breast cancer: real-world data from Japan. Anticancer Res. (2026) 46:293–300. doi: 10.21873/anticanres.17943 41469132

[33] Xu L Xie Y Gou Q . HER2-targeted therapies for HER2-positive early-stage breast cancer: present and future. Front Pharmacol. (2024) 15:1446414. doi: 10.3389/fphar.2024.1446414 39351085 PMC11439691

[34] Goorts B Nijnatten TJ Munck L . Clinical tumor stage is the most important predictor of pathological complete response rate after neoadjuvant chemotherapy in breast cancer patients. Breast Cancer Res Treat. (2017) 163:83–91. doi: 10.1007/s10549-017-4155-2 28205044 PMC5387027

[35] Hung CC Tsai IC Hsu CY Lin HC . Clinical outcomes of neoadjuvant therapy in human epidermal growth factor receptor 2 breast cancer patients: a single-center retrospective study. J Clin Med. (2022) 11:1434. doi: 10.3390/jcm11051434 35268525 PMC8911223

[36] Peng DM Li J Qiu JX Zhao L . Neoadjuvant pertuzumab plus trastuzumab in combination with chemotherapy for human epidermal growth factor receptor 2 positive breast cancer: a real-world retrospective single-institutional study in China. World J Surg Oncol. (2024) 22:88. doi: 10.1186/s12957-024-03365-x 38582875 PMC10998413

[37] Hännikäinen EN Mattson J Karihtala P . Predictors of successful neoadjuvant treatment in HER2 positive breast cancer. Oncol Lett. (2023) 26:434. doi: 10.3892/ol.2023.14021 PMC10472020 37664661

[38] Verdial FC Mamtani A Pawloski KR . The effect of age on outcomes after neoadjuvant chemotherapy for breast cancer. Ann Surg Oncol. (2022) 29:3810–9. doi: 10.1245/s10434-022-11367-w 35246810 PMC10901180

[39] Cortazar P Zhang L Untch M Mehta K Costantino JP Wolmark N . Pathological complete response and long-term clinical benefit in breast cancer: the CTNeoBC pooled analysis. Lancet. (2014) 384:164–72. doi: 10.1016/s0140-6736(13)62422-8 24529560

[40] Yang ZJ Xin F Chen ZJ Yu Y Wang X Cao XC . Real-world data on neoadjuvant chemotherapy with dual-anti HER2 therapy in HER2 positive breast cancer. BMC Cancer. (2024) 24:134. doi: 10.1186/s12885-024-11871-0 38273267 PMC10811850

[41] Ma X Zhang X Zhou X . Real-world study of trastuzumab and pertuzumab combined with chemotherapy in neoadjuvant treatment for patients with HER2-positive breast cancer. Med (Baltimore. (2022) 101:e30892. doi: 10.1097/md.0000000000030892 36221359 PMC9543020

[42] Medina EAG Caballero BB Miguel KL . Neoadjuvant trastuzumab and pertuzumab in combination with standard chemotherapy for HER2-positive early breast cancer: real-world practice in Cuba. Cancer Treat Res Commun. (2023) 34:100670. doi: 10.1016/j.ctarc.2022.100670 36549232

[43] Goutsouliak K Veeraraghavan J Sethunath V . Towards personalized treatment for early stage HER2-positive breast cancer. Nat Rev Clin Oncol. (2020) 17:233–50. doi: 10.1038/s41571-019-0299-9 31836877 PMC8023395

[44] Ji JH Bae SJ Kim SG . Anaemia and pathologic complete response rate according to carboplatin dose in HER2+ breast cancer treated with neoadjuvant TCHP. Cancer Med. (2023) 12:1409–17. doi: 10.1002/cam4.5022 35837812 PMC9883435

