# Supplementary material for: Real-world response rates in patients with human epidermal growth factor receptor 2-positive breast cancer after neoadjuvant treatment using dual human epidermal growth factor receptor 2 blockade
Source: Front Oncol. 2026 Sep 9;16:1895998. doi: 10.3389/fonc.2026.1895998 (PMC13597254; doi:10.3389/fonc.2026.1895998)
Supplement: Supplementary file 1 [file Table1.docx]

Supplementary Material

1. **Supplementary Tables**

**Supplementary Table 1:** HER2 status

| **HER2 status by IHC** (28) | |
| --- | --- |
| 0 | No staining is observed  OR  Membrane staining that is incomplete, faint/barely perceptible, and in ≤10% of tumor cells |
| 1+ | Incomplete membrane staining that is faint/barely perceptible and in >10% of tumor cells |
| 2+ | Weak to moderate complete membrane staining observed in >10% of tumor cells |
| 3+ | Circumferential membrane staining that is complete, intense, and in >10% of tumor cells |
| **HER2 status by FISH** (8) | |
| HER2–0 | IHC score of 0 |
| HER2-negative | - IHC score 1+ - IHC score 2+ + FISH ratio ≥2 + *HER2* gene copy number <4 signals/cell - IHC score 2+ + FISH ratio <2 + *HER2* gene copy number 4–6 signals/cell - IHC score 2+ + FISH ratio <2 + *HER2* gene copy number <4 signals/cell |
| HER2-positive | - IHC score 3+ - IHC score 2+ + FISH ratio ≥2 + *HER2* gene copy number ≥4 signals/cell - IHC score 2+ + FISH ratio <2 + *HER2* gene copy number ≥6 signals/cell |

*FISH ratio=HER2/CEP17 ratio.*

***CEP17:*** *Chromosome enumeration probe 17;* ***FISH:*** *Fluorescence in situ hybridization;* ***HER2:*** *Human epidermal growth factor receptor 2;* ***IHC:*** *Immunohistochemistry.*

**Supplementary Table 2:** ER and PR status

| **Allred score*** (29) | |
| --- | --- |
| **Positive cells (%)** | **Proportion score** |
| 0 | 0 |
| <1 | 1 |
| 1–10 | 2 |
| 11–33 | 3 |
| 34–66 | 4 |
| ≥67 | 5 |
| **Intensity** | **Intensity score** |
| None | 0 |
| Weak | 1 |
| Intermediate/moderate | 2 |
| Strong | 3 |
| **ER status** | **Allred score** |
| ER-negative | 0–2 |
| ER-positive | 3–8 |
| **PR status** | **Allred score** |
| PR-negative | 0–2 |
| PR-positive | 3–8 |

**The ER Total Allred Score is based on the percentage of cells that stain positive by IHC for ER and the intensity of that staining. The PR Total Allred Score is based on the percentage of cells that stain by IHC for PR and the intensity of that staining.*

*The Allred Score is a method of quantifying ER and PR using both the intensity and the percentage of positive cells. The Allred Score is calculated by adding the Proportion Score and the Intensity Score, as outlined in the table. The Allred score combines the percentage of positive cells (proportion score) and the intensity score of the reaction product in most carcinomas. The two scores are added together for a final score with eight possible values (00–08).*

***ER:*** *Estrogen receptor;* ***IHC:*** *Immunohistochemistry;* ***PR:*** *Progesterone receptor.*

**Supplementary Table 3:** pCR status

| **Response assessment** (30) | |
| --- | --- |
| pCR | The absence of residual invasive cancer in the breast or axillary lymph nodes (ypT0ypN0) |

*pCR: Pathological complete response.*

**Supplementary Table 4:** CTCAE v6.0 (31)

| **Toxicity** | **Grade 1** | **Grade 2** | **Grade 3** | **Grade 4** | **Grade 5** |
| --- | --- | --- | --- | --- | --- |
| Anemia | Hb <LLN – 10.0 g/dL;  <LLN – 6.2 mmol/L; <LLN – 100 g/L | Hb <10.0–8.0 g/dL;  <6.2–4.9 mmol/L;  <100–80 g/L | Hb <8.0 g/dL; <4.9 mmol/L;  <80 g/L; transfusion indicated | Life-threatening consequences;  urgent intervention indicated | Death |
| Neutropenia | - | - | ANC <1000/mm^3^ with a single temperature of >38.3°C (101°F) or a sustained temperature of ≥38°C (100.4°F) for more than 1 hour | Life-threatening consequences;  urgent intervention indicated | Death |
| Thrombocytopenia | <LLN – 75,000/mm^3^; <LLN – 75.0×10^9^/L | <75,000–50,000/mm^3^;  <75.0–50.0×10^9^/L | <50,000–10,000/mm^3^;  <50.0–10.0×10^9^/L;  transfusion indicated | <10,000/mm^3^; 10.0×10^9^/L;  life-threatening consequences;  urgent intervention indicated | Death |
| Diarrhea | Change in consistency or  frequency | Increase of 4–6 stools per day over baseline; moderate increase in ostomy output compared with that at baseline;  limiting instrumental ADL or mild/moderate impact on age-appropriate normal daily activity (pediatric);  change in consistency or frequency AND limiting instrumental ADL or mild/moderate impact on age-appropriate normal daily activity (pediatric) | Increase of ≥7 stools per day over baseline;  hospitalization indicated;  severe increase in ostomy output compared with that at baseline;  requires IV intervention;  limiting self-care ADL or severe impact on age-appropriate normal daily activity (pediatric) | Life-threatening consequences; urgent intervention indicated | Death |
| Fatigue | Fatigue relieved by rest | Fatigue not relieved by rest;  limiting instrumental ADL or mild/moderate impact on age-appropriate normal daily activity (pediatric) | Fatigue not relieved by rest,  limiting self-care ADL or severe impact on age-appropriate normal daily activity (pediatric) | - | - |
| Vomiting | Intervention not indicated | Initiation of outpatient IV  hydration;  medical intervention indicated | Initiation of tube feeding, or TPN;  hospitalization indicated | Life-threatening consequences | Death |
| Lung toxicity | Asymptomatic or mild symptoms; clinical or diagnostic observations only; intervention not indicated | Moderate; minimal, local, or noninvasive intervention indicated; limiting instrumental ADL or mild/moderate impact on age-appropriate normal daily activity (pediatric) | Severe or medically significant but not immediately life-threatening; hospitalization or prolongation of existing hospitalization indicated; limiting self-care ADL or severe impact on age-appropriate normal daily activity (pediatric) | Life-threatening consequences; urgent intervention indicated | Death |
| Cardiac toxicity | Asymptomatic or mild symptoms; clinical or diagnostic observations only; intervention not indicated | Moderate; minimal, local, or noninvasive intervention indicated; limiting instrumental ADL or mild/moderate impact on age-appropriate normal daily activity (pediatric) | Severe or medically significant but not immediately life-threatening; hospitalization or prolongation of existing hospitalization indicated; limiting self-care ADL or severe impact on age-appropriate normal daily activity (pediatric) | Life-threatening consequences; urgent intervention indicated | Death |
| **Hepatic toxicity** | Asymptomatic or mild symptoms; clinical or diagnostic observations only; intervention not indicated | Moderate; minimal, local, or noninvasive intervention indicated; limiting instrumental ADL or mild/moderate impact on age-appropriate normal daily activity (pediatric) | Severe or medically significant but not immediately life-threatening; hospitalization or prolongation of existing hospitalization indicated; limiting self-care ADL or severe impact on age-appropriate normal daily activity (pediatric) | Life-threatening consequences; urgent intervention indicated | Death |
| ALT | >ULN – 3.0×ULN | >3.0–5.0×ULN | >5.0–20.0×ULN | >20.0×ULN | - |
| AST | >ULN – 3.0×ULN | >3.0–5.0×ULN | >5.0–20.0×ULN | >20.0×ULN | - |
| Bilirubin | >ULN – 1.5×ULN | >1.5–3.0×ULN | >3.0–10.0×ULN | >10.0×ULN | - |
| ALP | >ULN–2.5×ULN | >2.5–5.0×ULN | >5.0–20.0×ULN | >20.0×ULN | - |
| GGT | >ULN–2.5×ULN | >2.5–5.0×ULN | >5.0–20.0×ULN | >20.0×ULN | - |
| Renal toxicity | Asymptomatic or mild symptoms; clinical or diagnostic observations only; intervention not indicated | Moderate; minimal, local, or noninvasive intervention indicated; limiting instrumental ADL or mild/moderate impact on age-appropriate normal daily activity (pediatric) | Severe or medically significant but not immediately life-threatening; hospitalization or prolongation of existing hospitalization indicated; limiting self-care ADL or severe impact on age-appropriate normal daily activity (pediatric) | Life-threatening consequences; urgent intervention indicated | Death |

***ADL:*** *Activities of daily living;* ***ALP:*** *Alkaline phosphatase;* ***ALT:*** *Alanine aminotransferase;* ***ANC:*** *Absolute neutrophil count;* ***AST:*** *Aspartate aminotransferase;* ***CTCAE:*** *Common Terminology Criteria for Adverse Events;* ***GGT:*** *Gamma-glutamyl transferase;* ***Hb:*** *Hemoglobin;* ***IV:*** *Intravenous;* ***LLN:*** *Lower limit of normal;* ***TPN:*** *Total parenteral nutrition;* ***ULN:*** *Upper limit of normal.*

**Supplementary Table 5:** Association between clinical factors and pCR in SC dual HER2 blockade and IV dual HER2 blockade groups

| **Parameter** | **pCR** | | **Overall** | **p value** |
| --- | --- | --- | --- | --- |
|  | **Yes** | **No** |  |  |
| **SC dual HER2 blockade (N=52)** | | | | |
| **Age (years)** |  |  |  |  |
| ≤50 | 7 (13.5) | 5 (9.6) | 12 (23.1) | 0.329 |
| >50 | 16 (30.8) | 24 (46.2) | 40 (76.9) |  |
| **Menopausal status** |  |  |  |  |
| Postmenopausal | 16 (30.7) | 25 (48.1) | 41 (78.8) | 0.1822 |
| Premenopausal | 7 (13.5) | 4 (7.7) | 11 (21.2) |  |
| **Composite clinical stage** |  |  |  |  |
| I–II | 17 (32.7) | 10 (19.2) | 27 (51.9) | **0.0108** |
| III–IV | 6 (11.5) | 19 (36.6) | 25 (48.1) |  |
| **ER status** |  |  |  |  |
| Positive | 12 (23.1) | 21 (40.3) | 33 (63.4) | 0.2242 |
| Negative | 11 (21.2) | 8 (15.4) | 19 (36.6) |  |
| **Ki-67 index (%)** |  |  |  |  |
| <20 | 0 (0) | 2 (3.8) | 2 (3.8) | 0.4969 |
| ≥20 | 23 (44.3) | 27 (51.9) | 50 (96.2) |  |
| **IV dual HER2 blockade (N=22)** | | | | |
| **Age (years)** |  |  |  |  |
| ≤50 | 3 (13.6) | 3 (13.6) | 6 (27.3) | 0.334 |
| >50 | 12 (54.5) | 4 (18.2) | 16 (72.7) |  |
| **Menopausal status** |  |  |  |  |
| Postmenopausal | 11 (50.0) | 3 (13.6) | 14 (63.6) | 0.3426 |
| Premenopausal | 4 (18.2) | 4 (18.2) | 8 (36.4) |  |
| **Composite clinical stage** |  |  |  |  |
| I–II | 8 (36.4) | 3 (13.6) | 11 (50.0) | 0.9999 |
| III–IV | 7 (31.8) | 4 (18.2) | 11 (50.0) |  |
| **ER status** |  |  |  |  |
| Positive | 6 (27.3) | 4 (18.2) | 10 (45.5) | 0.6517 |
| Negative | 9 (40.9) | 3 (13.6) | 12 (54.5) |  |
| **Ki-67 index* (%)** |  |  |  |  |
| <20 | 1 (5.3) | 0 (0) | 1 (5.3) | 0.999 |
| ≥20 | 11 (57.9) | 7 (36.8) | 18 (94.7) |  |

*Data are presented as n (%).*

*For SC dual HER2 blockade, percentages are calculated with 52 (total number of patients in the SC dual HER2 blockade group) as the denominator.*

*For IV dual HER2 blockade, percentages were calculated with 22 (total number of patients in the IV dual HER2 blockade group) as the denominator.*

**Values have been calculated from data available for 19 patients, with 19 as the denominator.*

***ER:*** *Estrogen receptor;* ***HER2:*** *Human epidermal growth factor receptor 2;* ***IV:*** *Intravenous;* ***Ki-67:*** *Kiel 67;* ***pCR:*** *Pathological complete response;* ***SC:*** *Subcutaneous.*
